# Identification of *Cocconeis neothumensis* var. *marina* using a polyphasic approach including ultrastructure and gene annotation

**DOI:** 10.1371/journal.pone.0317360

**Published:** 2025-02-13

**Authors:** Emanuele Somma, Maria Costantini, Chiara Pennesi, Nadia Ruocco, Olga De Castro, Antonio Terlizzi, Valerio Zupo

**Affiliations:** 1 Ischia Marine Centre, Department of Ecosustainable Marine Biotechnology, Stazione Zoologica Anton Dohrn, Ischia (NA), Italy; 2 Department of Life Science, University of Trieste, Trieste, Italy; 3 Department of Ecosustainable Marine Biotechnology, Stazione Zoologica Anton Dohrn, Naples, Italy; 4 Department of Integrative Marine Ecology, Stazione Zoologica Anton Dohrn, CRIMAC, Calabria Marine Centre, Amendolara (CS), Italy; 5 Department of Ecosustainable Marine Biotechnology, Stazione Zoologica Anton Dohrn, Calabria Marine Centre, Amendolara (CS), Italy; 6 Department of Biology, University of Naples Federico II, Naples, Italy; 7 Botanical Garden, University of Naples Federico II, Naples, Italy; 8 Department of Integrative Marine Ecology, Stazione Zoologica Anton Dohrn, Naples, Italy; 9 NBFC, National Biodiversity Future Center, Palermo, Italy; National Cheng Kung University, TAIWAN

## Abstract

Several microalgae, including marine diatoms, significantly contribute to the global primary production and play a vital role in the food webs of benthic and planktonic ecosystems. Diatoms of the genus *Cocconeis* frequently inhabit benthic substrates, including the leaves of seagrasses. They are seasonally dominant in the leaf epiphytic layer of the Mediterranean seagrass *Posidonia oceanica* L. Delile, and have been proposed as model organisms for chemical ecology studies. However, the genome of *Cocconeis* spp. has not been sequenced. Consequently, their low-level molecular identification is currently impossible, besides a few examples. To address this gap, a polyphasic identification of *C. neothumensis* has been employed, combining ultra-morphological data with DNA barcoding markers. A strain of diatoms was isolated from *P. oceanica* leaves. It has been cultured in the laboratory and examined under Scanning Electron Microscopy (SEM). The 18S ribosomal RNA gene (18S rRNA, nrDNA) and the ribulose 1,5-biphosphate carboxylase *(rbc*L, cpDNA) gene were analysed for DNA barcoding characterisation. Since ultra-morphology data unambiguously identified the isolated strain as *C. neothumensis* Krammer, 1991, the molecular sequences herein reported will facilitate its rapid and accurate identification. In addition, our comparative analyses will facilitate the evaluation of these molecular markers for identification of closely related benthic diatoms.

## Introduction

Diatoms (Bacillariophyceae) are photosynthetic eukaryotic organisms included into a wide and diverse taxonomic group that may become dominant both in benthic and planktonic environments [1]. Diatoms may play crucial ecological roles, by promoting a high productivity [2] due to a fast turnover [3] and assimilating more than 20% of the atmospheric CO_2_, so contributing to retard Ocean Acidification (OA) processes [4]. They are autotrophic unicellular organisms, but in some special conditions they may exhibit a partially heterotrophic metabolism. Diatoms may be organized as individual cells or be colonial. They may be found at almost any aquatic habitat. Some species may be symbionts of invertebrates and in specific cases they influence the physiology of marine arthropods [1] upon ingestion, or their behaviour (Jüttner et al., 2010) through the emission of volatile organic compounds (VOCs). Since they are characterised by a typical and unique double-capsule silica shell (frustules), several biotechnological applications have been set in the frame of microfiltration or the production of micro-structures [5].

In addition, diatoms produce interesting bioactive compounds [6] and many studies in the last decades explored the potential of planktonic and benthic diatoms to produce ecologically bioactive compounds [7] or substances with anti-cancer and other medical purposes [8]. Diatoms are also characterised by a typical peroxidase biosynthetic pathway [9], generally wound-activated, leading to the production of volatile compounds (VOCs) and lipidic molecules exhibiting specific biological activities [10]. They also have interesting biotechnological potentials [11]. Transcriptomic investigations on a common diatom (*Nitszchia* sp., which produces up to 50% oil by weight, under defined conditions) indicated that environmental influences, such as increased salinity, induce a higher content of total lipids, due to a boosted biosynthesis of triacylglycerols [12].

For these reasons, the biological effects of several species of diatoms have been analysed [13] and some of them have been submitted to molecular investigations. The genome of *Phaeodactylum* (*Phaeodactylon*) *tricornutum* Bohlin (1897) was sequenced [14], showing that it is made of about 27.4 mega-bases and contains 10,402 genes. These studies revealed about 130,000 expressed sequenced tags (ESTs), which were further investigated and annotated [15]. However, a high-quality genome annotation is still missing for most marine diatoms [16]. This is a major obstacle to understand key molecular and cellular processes, important for ecological relationships and biotechnological applications. In addition, DNA fingerprinting is increasingly adopted to speed-up the identification of diatoms and produce timely answers to blooms and other phenomena influencing the ecological status of coastal waters [17].

The shape of taxonomically important microstructures, such as silica frustules, has been historically used for the morphological determination of species [18] through Scanning Electron Microscopy (SEM) observations. This process includes the evaluation of length, width, striation patterns and allometric shape, as described by the first axis, in a morphometric analysis. However, the morphological identification of diatoms is challenging, especially when intra-genus differences must be revealed [19]. Molecular tools complementing the morphological identification permit to precisely estimate their biodiversity. Molecular analyses based on DNA sequencing of specific ribosomal genes have been developed for classification purposes, and they are increasingly applied to facilitate the identification of diatoms, classically based on ultra-structural characters. However, a polyphasic approach, incorporating morphological and molecular data, guarantees for the accuracy of assessments. In fact, molecular-assisted taxonomy, combining molecular delimitation of species with *post-hoc* morphological examinations, was proven effective for the classification of congeneric diatoms, often characterised by similar ultra-morphology.

Molecular tools for the identification of diatoms are available for a limited number of species, primarily for key planktonic taxa, while annotated genomes remain unavailable for most benthic species [20]. Molecular markers are currently available at the National Center for Biotechnology Information (NCBI) database for a few species of the genus *Cocconeis*, (namely *Cocconeis placentula*, *Cocconeis stauroneiformis*, *Cocconeis euglypta*, and *Cocconeis pediculus*). In fact, a species-level identification by molecular tools is impossible for such diatoms as *C. neothumensis*, notwithstanding their demonstrated importance for the ecology of seagrass meadows [21,22] and the role played in important benthic environments [23]. Several investigations related to the Mediterranean region were carried out during the last decade, providing descriptions of several new species of *Cocconeis*. Despite of this evidence, most species were described only for their type localities, while information on their actual range of distribution is still missing [22]. *Posidonia oceanica* meadows, in particular, showed to be important reservoirs of diatom genetic diversity and *Cocconeis* spp. are often dominant in these environments.

*Cocconeis neothumensis* Krammer, 1991, in turn, is a model diatom [24] adopted in ecological investigations, and it is a source of bioactive compounds [6]. Its extracts exhibited promising activity for biotechnological applications in the fields of aquaculture and human medicine [25]. Some congeneric species living on leaves of seagrasses showed similar activities [26]. This study was aimed at producing for the first time monoclonal axenic cultures of *Cocconeis neothumensis* and provide a full taxonomical identification by means of SEM ultra-morphology observations and annotated 18S ribosomal RNA (*18S rRNA*, nrDNA) and ribulose 1,5-biphosphate carboxylase (*rbc*L, cpDNA) genes, in order to fill the lack of information about molecular phylogenesis and eDNA metabarcoding of *Cocconeis* spp.. Our results will provide new tools for taxonomists, and they will facilitate the identification of several *Cocconeis* species, representing seasonally important players in the ecology of benthic communities [27] and promising sources of bioactive compounds.

## Materials and methods

### Sampling and sites

Low-adhesion panels holding eight glass disks coated with a silicone film were assembled and deployed by scuba divers to collect benthic diatoms. Each panel consisted of a two-sided plastic frame, a mooring strap for anchoring and a buoy set to assure a vertical positioning (S1 Fig). Four glass disks (individual surface area of 23.75 cm^2^) were applied on each side of the panels to select the species with strongest adhesion power, such as the diatoms of the genus *Cocconeis*. This collection device was designed to remain in a vertical position while freely floating, so emulating the conditions of seagrasses leaves, which stand vertically and receive epiphytic colonisers. The collecting panels were anchored at 5 m depth, nearby *P. oceanica* meadows*,* in autumn (October), using metal sticks. Two *P. oceanica* meadows were selected off the island of Ischia (Bay of Naples, Italy), at Cartaromana bay and Sant’Anna rocks (S2 Fig), for one month of exposure, prior to collect the panels and start the isolation of diatoms. All sampling operations were performed within the Marine Protected Area (MPA) “*Neptune Kingdom*”, after obtaining a formal approval of the MPA Director for access and collection.

### Diatom isolation and culture

In the laboratory, the coated low-adhesion slides were individually removed from the panels, rinsed with filtered seawater (0.22 µm TPP vacuum filtration “rapid” - Filtermax) to reduce the presence of scarcely adhesive items, and gently scraped with a sterile glass slide to collect the most adhesive colonizers (including *Cocconeis* spp. diatoms). The epiphytes were transferred to Petri dishes and analysed under an inverted microscope to isolate diatoms belonging to the genus *Cocconeis*. To this end, each sample was divided into 6 sub-samples, each one transferred to a 4 cm well containing 6 mL of filtered seawater. The diatoms of interest were isolated under a Leica inverted microscope by collecting single cells with a micromanipulator (Leica Microsystems, Milan, Italy) equipped with a Narishige syringe (Narishige International Ltd., London, UK).

Individually collected diatoms were transferred to a sterile well containing 6 mL of autoclaved seawater and monitored every other day under an inverted microscope to track their proliferation and ensure the absence of contaminant organisms. The most promising strains were transferred again, while those demonstrating contamination or absence of growth were rejected. Once monoclonal strains were obtained, they were transferred into Guillard’s *f/2* medium with silicates (Sigma-Aldrich, Milan, Italy). Transfers and renewals of strains were carried out under a laminar flow hood using sterilised glassware. Diatom cultures were kept in 6-well plastic multi-wells in a thermostatic chamber (18 °C; 12:12 h light:dark). Light was provided by a Sylvania GroLux lamp (Osram Sylvania Inc., USA) assuring an irradiance of 140 μE m^−2^ s^−1^. Cultures were renewed under laminar flow hood by transferring a few cells (scraped by a sterile glass pipette) in freshly prepared *f/2* every 15 days.

### Methods of preparation and morphological identification

Diatoms were initially identified using a morphological approach, by analysing their ultrastructure under SEM (SEM Philips XL20, USA). Two stubs were prepared for each mother-culture by collecting a portion of diatom film from the wells. Each sample was transferred into glass centrifuge tubes to undergo an acid-cleaning protocol for the removal of the organic matter. The von Stosch (1974) method was applied, modified for the specific purposes of this study. In particular, samples were rinsed with distilled water and centrifuged three times (2,504 g) for 10 minutes. The supernatant was withdrawn and replaced with distilled water following each centrifuge cycle. After the removal of most supernatant, the volume of the sample was measured and an equal volume of 65% HNO_3_ was added and kept 60 minutes at 60 °C in a stove. Following this step, the volume of H_2_SO_4_ was triplicated and its concentration reached 98%. Glass tubes were then moved upon the reducing flame of a Bunsen burner until the pellet started to produce bubbles. Following this procedure, the organic matter was totally degraded while the siliceous frustules remained intact. Further, the pellets were centrifuged and rinsed several times with distilled water, until neutral pH was reached. Finally, they were dehydrated with 90% EtOH. Stubs were prepared by applying a double-sided tape on the stub covered with a 0.5 μm filter (Millipore^TM^, Isopore membrane filters). After resuspension of the pellet in EtOH, a drop of the suspension was placed on the filter. The stubs were dried 24 hours under a hood to be ready for SEM observations. The frustule morphology was described according to Hustedt [28], Krammer [29], and De Stefano et al. [21].

### DNA barcoding identification

The same strains analysed for SEM were subjected to molecular identification. To this purpose, a small number of diatoms were collected from the bottom of the wells using a sterile Pasteur pipette and used for amplification of partial sequences of both *18S* rRNA and *rbcL* genes from nuclear and plastid DNA, respectively. Several tests were conducted to optimise the lysis process on diatom cells using different lysis buffers for genomic DNA extractions, because diatom cells are known to be recalcitrant to disruption [30]. The Lyses & Bacteria/Fungi PCR-GO Kit procedure (DNATech srl, Naples, Italy), equivalent to other commercially available kits (e.g., PicoPure™ DNA Extraction Kit, Applied Biosystems), was used to perform PCR from single cells following the method proposed by Carraturo et al. [31]. Briefly, the cells collected using a Pasteur pipette were resuspended and lysed with 20 μL of denaturation lysis buffer at 98 °C for 10 minutes. After centrifugation (> 15650 g for 5 minutes), the supernatant was diluted 1:5, and 1 μL was used for direct PCR amplification using Phire Hot Start II Polymerase (Thermo Fisher Scientific, Waltham, MA, USA), following the manufacturer’s instructions.

The PCR was performed using primers for diatoms: (i.) 528F, 5’GCG GTA ATT CCA GCT CCA A-3’ and 1055R, 5’ACG GCC ATG CAC CAC CAC CCA T-3’, amplifying a 18S rRNA fragment of 800 bp [32,33]; and (ii.) rbcL-F, 5’-ATG TCT CAA TCT GTA WCA GAA CGG ACT C-3’ and rbcL-R, 5’-TAA RAA WCK YTC TCT CCA ACG CA-3’, amplifying a *rbc*L fragment of 660 bp [34,35]. The amplified fragments were purified from an agarose gel (1.5%) using the *QIAquick Gel Extraction kit* (Qiagen, Milan, Italy). The purified amplicons were sequenced using both forward and reverse primers with the BigDye® Terminator v3.1 Cycle Sequencing kit (Life Technologies, Thermo Fisher Scientific, Waltham, MA, USA). The reactions were then analysed by an Applied Biosystems 3730 DNA Analyzer (Life Technologies, Thermo Fisher Scientific, Waltham, MA, USA). The obtained electropherograms were edited, assembled and aligned using *MultiAlin* software (http://multalin.toulouse.inra.fr/multalin/) [36]. To assess their taxonomical identity, the sequences were compared against the nucleotide collection available in the public database using BLASTn (https://blast.ncbi.nlm.nih.gov; accessed on January 2024). Multiple alignments of the sequences and the related trees maximum likelihood phylogenetic tree, based on sequences of the *18S rRNA* and *rbcL* genes, were performed by Clustal W (https://www.genome.jp/tools-bin/clustalw; PhyML bootstrap). Sequences were deposited to GenBank (accession number PQ740503; https://ncbi.nlm.nih.gov/genbank).

## Results

### Isolation of pure strains

Twenty-six pure strains of diatoms were obtained after two months of successive isolations and 14 of them contained *Cocconeis* spp., while others contained *Mastogloia* spp. and other genera belonging to the type Pennales, not yet analysed under SEM microscopy. In particular, the strain named *CnN* (presently available in a live culture at the Stazione Zoologica Anton Dohrn) was further analysed according to the procedures above described, to reach a polyphasic identification. The mother culture is renewed every 16–20 days, following the method described by Raniello et al. [24].

### Description of the species

The identification conducted using the Scanning Electron Microscopy images led to the following determination of the strain *CnN.*

***Cocconeis neothumensis* var. *marina*** M. De Stefano et al. [21]

Order: Achnanthales P. Silva

Family: Cocconeidaceae Kützing

Genus: *Cocconeis* Ehrenberg

*Reference*. De Stefano et al. [21] (p. 233, refer to the Figures 53–65 of the original paper)

Material examined. Specimens were collected at Sant’Anna Rock (40°43′34.68″ N, 13°57′40.92″ E).

**Fig 1:**
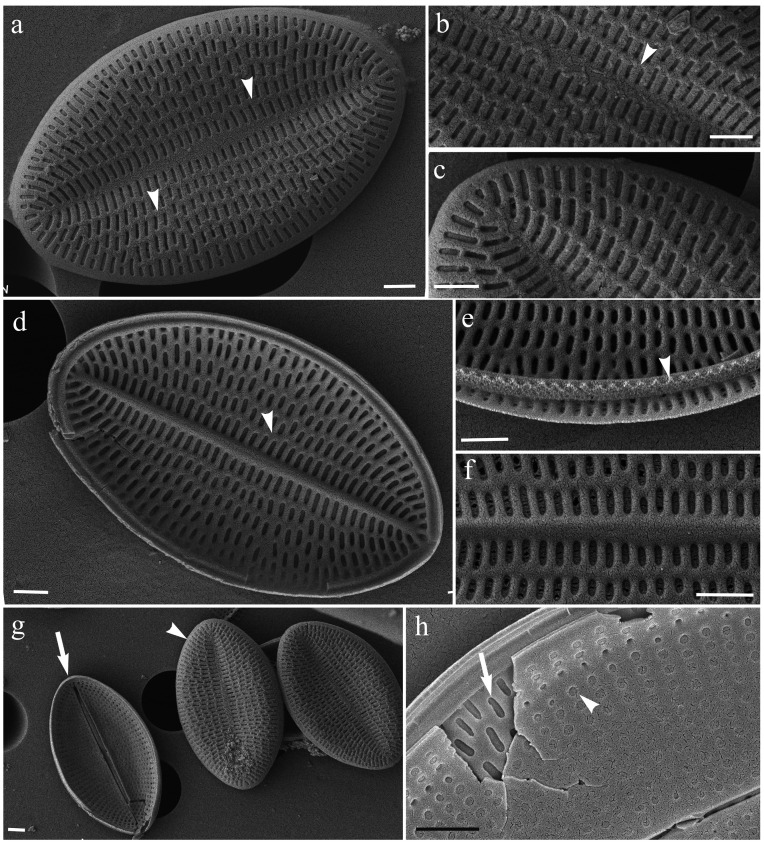
*Cocconeis neothumensis* var. *marina*, SEM. (a) External SV view showing shallow depression; (b) External SV view showing longitudinal ribs in the central valve zone (arrowhead); (c) External SV view of apex with transapical striae; (d) Internal SV view showing longitudinal ribs (arrowhead); (e) Broken cingulum (arrowhead); (f) Focus on SV areolae; (g) Panoramic on valves: SV (arrowhead) and RVS (arrow); (h) Broken frustule showing the different areolae between RSV (rounded areolae; arrow) and SV (rectangular areolae; arrowhead). Scale bars: 1 μm.

Description. The valves are lanceolate to elliptical-lanceolate almost circular with rounded apices (Figs 1, a d, g; and 2, a, c, d). The length varies from 14.2 to 10.8 μm, and the width from 8.3 to 6 μm. Transapical striae are radiate, from 20 to 30 in 10 μm.

**Fig 2:**
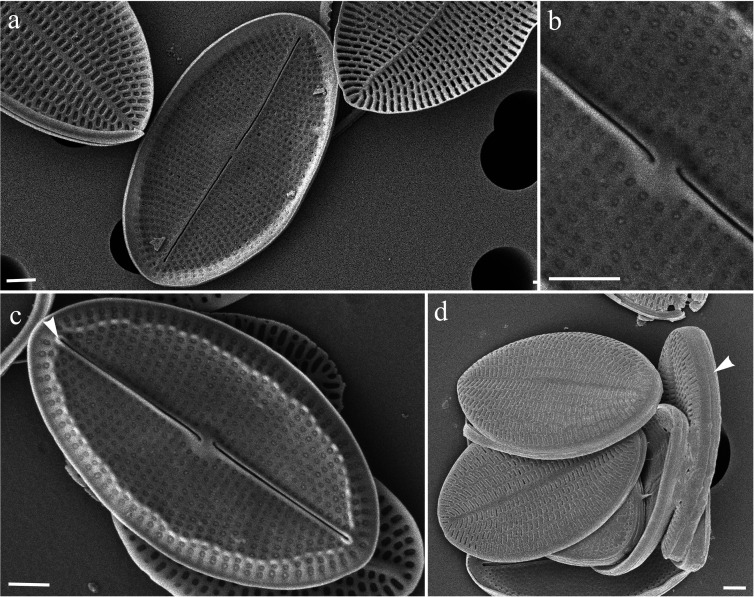
*Cocconeis neothumensis* var. *marina*, SEM. (a) External RSV view showing the straight branches of the raphe. (b) Internal RSV view showing the central nodule. (c) Internal RSV view showing raphe with small helictoglossae (arrowhead). Scale bars: 1 μm.

Sternum valve (SV). Externally the valve is centrally slightly depressed toward the thin, straight sternum (Fig 1a, g). Longitudinal ribs are visible both externally and internally in valve view (Fig 1, a, b, d, arrowheads).

Raphe-sternum valve (RVS). Externally, the raphe consists of two straight branches ending centrally and distally in apically expanded pores (Fig 2a). Internally, the straight raphe branches are bordered by ribs, ending centrally in simple pores gently bent toward the opposite side (Fig 2, b, c). The central nodule and the small helictoglossae at the poles are visible (Fig 2c, arrowhead).

### DNA barcoding identification

**Fig 3:**
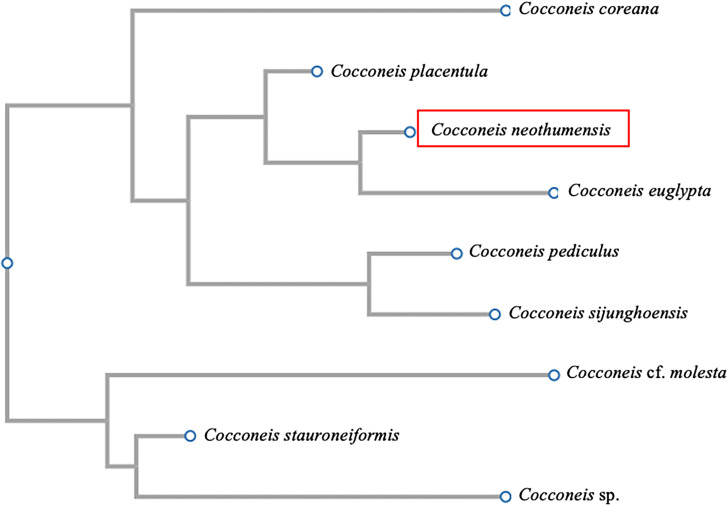
Maximum likelihood phylogenetic tree based on sequences of the 18S rRNA gene. The analysis includes *Cocconeis* sp. (HG993259), *Cocconeis coreana* (LR890011), *Cocconeis euglypta* (KM592933), *Cocconeis* cf. *molesta* (AJ535148), *Cocconeis pediculus* (FR873235.1*), Cocconeis placentula* (FR873239), *Cocconeis sijunghoensis* (KM592930), *Cocconeis stauroneiformis* (AB430614) and *Cocconeis neothumensis* var. *marina* identified in the present study (highlighted with the red box) (Clustal W, https://www.genome.jp/tools-bin/clustalw; PhyML; bootstrap only values above 50% are shown).

When the same strain, morphologically analysed as described above, was investigated to confirm its identification using molecular techniques, BLASTn analyses of the 18S rRNA gene confirmed the genus level identification obtained through SEM investigations and OM observations. The diatom displayed a high sequence similarity (97.9%) with the first hit, *C. placentula* (GenBank accession no.: AM502013). Close phylogenetic relationships with other annotated diatoms belonging to the genus *Cocconeis* were confirmed by the phylogenetic tree based on the 18S rRNA gene, using a maximum likelihood method for the estimation of relationships (Fig 3 and S3 Fig).

**Fig 4:**
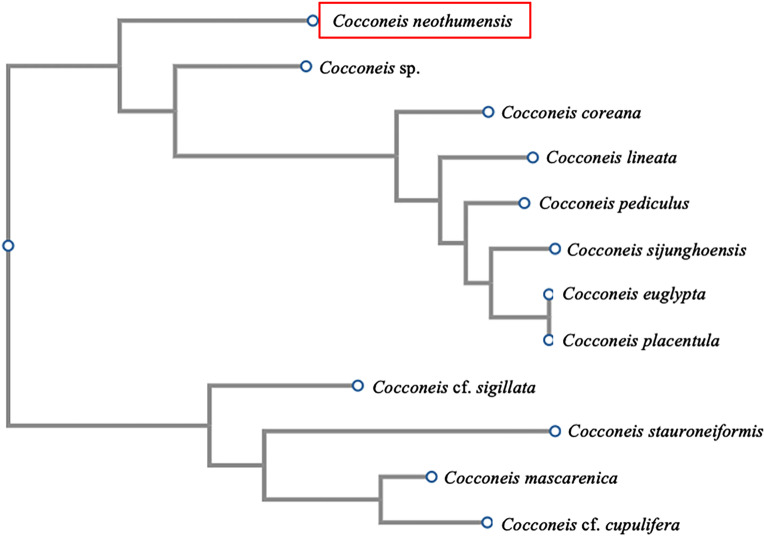
Maximum likelihood phylogenetic tree based on sequences of the *rbc*L gene. The analysis includes *Cocconeis* sp. (LR890022), *Cocconeis coreana* (LR890017), *Cocconeis* cf. *cupulifera* (KT943680), *Cocconeis euglypta* (KT072907), *Cocconeis lineata* (LR890020), *Cocconeis mascarenica* (MK454988), *Cocconeis pediculus* (KM084991), *Cocconeis placentula* (MW484810), *Cocconeis* cf. *sigillata* (MT015687), *Cocconeis sijunghoensis* (LR890019.1), *Cocconeis stauroneiformis* (AB430694) and *Cocconeis neothumensis* var. *marina* identified in the present study (highlighted with the red box). (Clustal W, https://www.genome.jp/tools-bin/clustalw; PhyML; bootstrap only values above 50% are shown).

According to the *rbc*L gene, the BLASTn analyses showed as the first hit the diatom *C. euglypta* (GenBank accession no.: LR890021) with 94.6% of pairwise sequence similarity. Similarly, when the phylogenetic relationship with other annotated diatoms belonging to the *Cocconeis* genus was analysed, based on the *rbc*L gene (Fig 4), *C. neothumensis* was identified and found to be closely related to other congeneric species (S4 Fig).

## Discussion

### Morphological identification of adhesive diatoms

Notwithstanding the long history of taxonomic studies performed on benthic diatoms, the difficulties associated with identification and classification of diatoms are still a major challenge, mainly due to their small size and ultra-morphological similarities. In fact, several investigations avoided the identification at the species level, or dealt only with the community dynamics of unidentified benthic diatoms [37,38]. However, the precise identification of diatom strains is crucial for most studies, due to their specific ecological constraints, as well as the individual physiology and biochemistry characterising each species [39]. The isolation of the *CnN* strain represents a significant achievement, because benthic diatoms are much more difficult to sample and quantify than planktonic ones, because of their strong adhesion to substrates.

To easily and quickly reveal the hidden diversity of adhesive diatoms, whose morphological identification is largely attributed to their tiny details, the development of molecular barcoding techniques and the construction of comprehensive genetic databases [40,41] is urgently needed. In fact, several researches investigated the ecology and the physiology of benthic diatoms characterised by “intermediate” adhesive power, such as *Skeletonema marinoi* [42], *Nanofrustulum shiloi* and *Striatella unipunctata* [43], whereas a few studies took into account alive strains of *Cocconeis* spp. [20,44] and *Mastogloia* spp. [45,46]. This is due to their strong adhesive power and also to the delicate organization of their frustules, which makes the process of isolation and culture of strains quite complex. Consequently, biologists and ecologists have largely disregarded these species of diatoms and only in the last decades some data have been made available [47–49], taking mainly into account the community assemblages and the general taxonomy of a small number of benthic diatoms [50].

The isolation and further culture of benthic diatoms with strong adhesive power (such as *Cocconeis* spp.) is complex and time-consuming, also because they grow slowly and are generally overwhelmed by the proliferation of other benthic species, less adhesive and mobile [51,52]. In our case, the process required the development of *ad-hoc* isolation techniques, coupled with the processes of sequential transfers, demanding daily evaluations of the isolated cells to ensure that the culture was not contaminated by other microorganisms. Following these steps, this task required huge efforts, up to the final isolation and characterisation. As well, the frustule cleaning method (proposed by Von Stosch [53], and followed by De Stefano et al., [21] was effective for *Cocconeis* spp. only after the modifications we developed (as in methods), because these diatoms exhibit slender frustules which are rapidly destroyed by warm acids. The modified technique led to a correct identification of the species, based on the morphology of frustules and the distribution of pores.

### Polyphasic approaches

Molecular identification methods [34] are mainly based on conserved DNA sequences, such as internal transcribed spacers (ITS), considered to be good molecular markers for phylogenetic analyses. They changed our understanding of conventional taxonomy [54]. However, partial subunits of ribosomal RNA genes (SSU rDNA) are also useful for this purpose. The *rbc*L gene was suggested [55] as the most appropriate molecular marker for the identification and the classification of benthic diatoms. The 18S rRNA, *rcb*L, along with the mitochondrial cytochrome c oxidase I (*cox*I) and the internal transcribed spacer (ITS) genes have been frequently used, because they are key genetic markers [56].

The strain of *C. neothumensis* var. *marina* herein described closely recalls the original diagnosis provided by De Stefano et al. [21], with the notable exception of the cell size. In fact, our specimens are slightly longer and wider than those observed by De Stefano et al. [21]. Moreover, the valves of the samples collected in Ischia for this study display a maximum of 30 longitudinal striae in the space of 10 μm, whereas De Stefano et al. [21] reported a maximum of 26 striae. Additionally, we showed for the first time the SEM images of the external SV (Figs 1 and 2), which reveal the depression and the longitudinal ribs which were previously visible only in a small LM image, in the original publication (please refer to the original Figure 53 by De Stefano et al., [21]).

The molecular techniques applied confirmed the results of the morphological identification, showing sequence similarity, with *C. placentula* as the first hit. Additionally, the rbcL gene alignment indicated *C. euglypta* as the first hit. In fact, *C. neothumensis* var. *marina* was never annotated in any repository or database [57,58] and its sequences were still unknown. Consequently, the molecular tests identified the closest species already annotated as the presumptive targets [59] and it is worth to note how this strain of *C. neothumensis* var. *marina* shares several morphological characters with the above species and their varieties [21].

Despite diatoms include at least 30,000 species (up to 100,000 according to [60]) the number of gene sequences deposited in GenBank is still quite small. Evidently, the completeness of a database has direct and absolute influence on the applicability and efficiency of the DNA barcoding techniques [61]. Currently, genetic information on most species of diatoms could not be found in GenBank, indicating that the database is still insufficient, and that molecular taxonomy studies on benthic diatoms are urgently needed.

The 18S rRNA gene is characterised by low resolution [57,58], being linked to the fact that *18S rRNA* gene of benthic diatoms has undergone unusually rapid evolutionary changes [62,63]. Thus, although the 18S rRNA has been widely used for diatom phylogenetic studies and has the largest database, with respect to other genetic markers, it could not solve all the identification uncertainties if not included into a polyphasic approach and using other molecular markers. Due to a relatively low sequence distance within a genus, the 18S rRNA might be not an appropriate genetic marker to differentiate diatom species, exhibiting a low resolution, insufficient to detect the polyphyletic characteristics of several species [64]. In addition, a previous study [55] showed that the *rbc*L gene represents a better molecular marker than 18S rRNA to identify and phylogenetically classify benthic diatoms. Consequently, the sequence identification herein provided, along with the techniques for isolation, cultivation and analysis of benthic diatoms, will be worth promoting the construction of a comprehensive data-base facilitating an easier identification of such adhesive species of benthic diatoms as *Cocconeis* spp., which play key ecological roles in the epiphytic compartments of seagrass meadows.

## Supporting information

S1 Fig Collection device. Schematic representation of the collection device for benthic diatoms (on the left). The frontal and lateral views of the panel bearing the low-adhesion glass slides are shown on the right side.
(PDF)

S2 Fig Sampling site. Sampling site located in the Cartaromana Bay—Sant’Anna rocks (40°43′34.68″ N, 13°57′40.92″ E) on the East coast of the Ischia Island (Gulf of Naples, Italy).
(PDF)

S3 Fig Alignment of *18S rRNA* gene. The sequences of the diatom *Cocconeis neothumensis* obtained in this work were aligned with the BLAST first hit *Cocconeis placentula* (Accession Number: AM502013.1), exhibiting 97.9% of pairwise sequence similarity.
(PDF)

S4 Fig Alignment of *rbcL* gene. The sequences of the diatom *Cocconeis neothumensis* obtained in this work were aligned with the BLAST first hit *Cocconeis euglypta* (Accession Number: LR890021.1) exhibiting 94.6% of pairwise sequence similarity.
(PDF)
