# Supplementary figures and images for: Identification of Cocconeis neothumensis var. marina using a polyphasic approach including ultrastructure and gene annotation
Source: PLoS One. 2025 Feb 13;20(2):e0317360. doi: 10.1371/journal.pone.0317360 (PMC11825096; doi:10.1371/journal.pone.0317360)

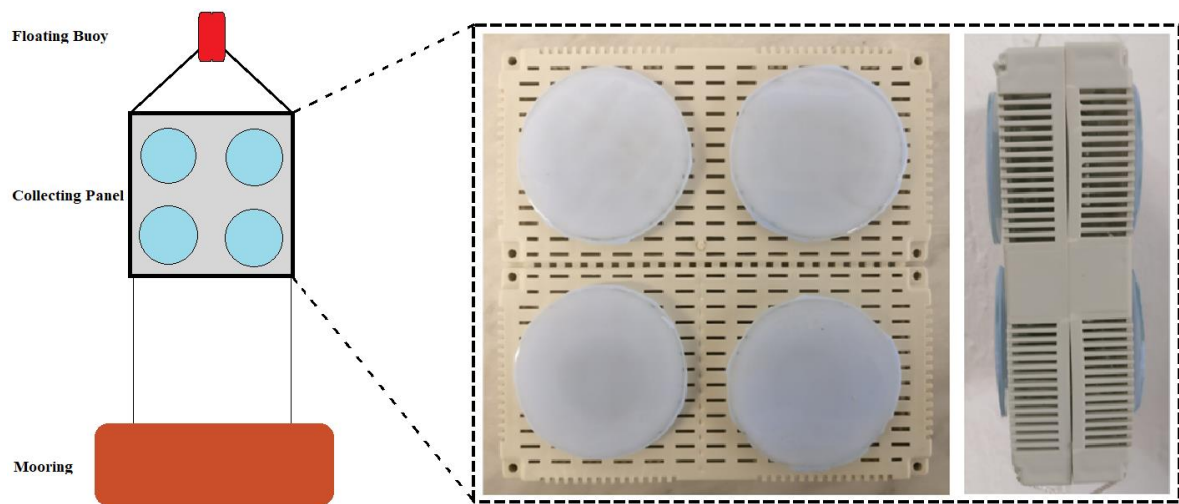

Supplement: S1 Fig — Schematic representation of the collection device for benthic diatoms (on the left). The frontal and lateral views of the panel bearing the low-adhesion glass slides are shown on the right side. (PDF) [file pone.0317360.s001.pdf]

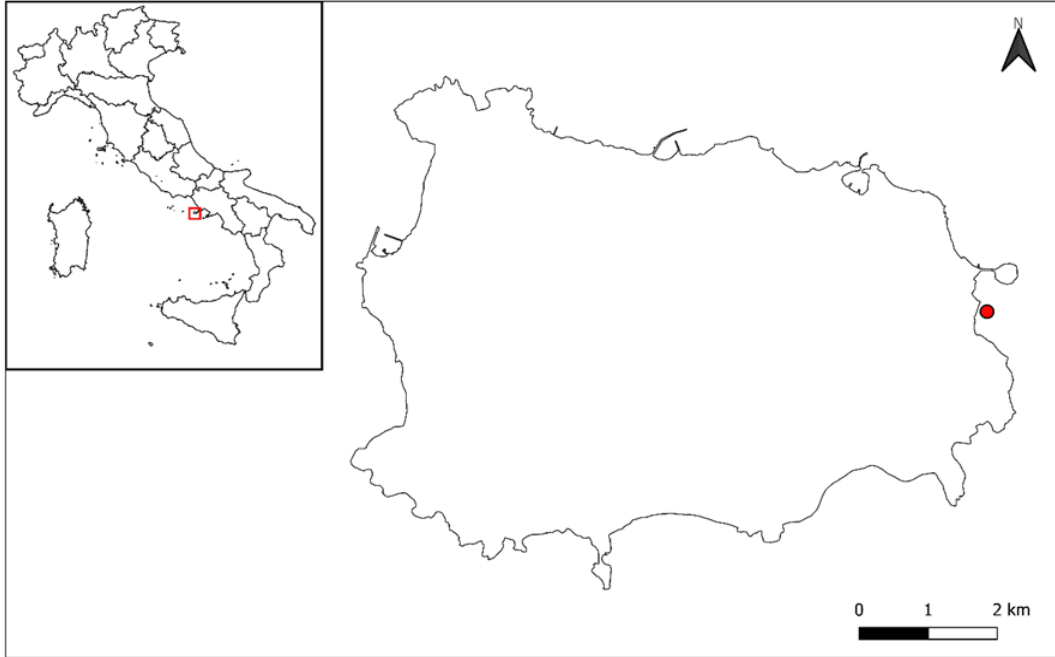

Supplement: S2 Fig — Sampling site located in the Cartaromana Bay—Sant’Anna rocks (40°43′34.68″ N, 13°57′40.92″ E) on the East coast of the Ischia Island (Gulf of Naples, Italy). (PDF) [file pone.0317360.s002.pdf]
