# Supplementary material for: Identification of Cocconeis neothumensis var. marina using a polyphasic approach including ultrastructure and gene annotation
Source: PLoS One. 2025 Feb 13;20(2):e0317360. doi: 10.1371/journal.pone.0317360 (PMC11825096; doi:10.1371/journal.pone.0317360)
Supplement: S3 Fig — The sequences of the diatom Cocconeis neothumensis obtained in this work were aligned with the BLAST first hit Cocconeis placentula (Accession Number: AM502013.1), exhibiting 97.9% of pairwise sequence similarity. (PDF) [file pone.0317360.s003.pdf]

```
                   1       10        20        30        40        50        60        70        80        90        100       110       120       130
                   |--------+---------+---------+---------+---------+---------+---------+---------+---------+---------+---------+---------+---------|
       AM502013.1  TAGTCATCCGCTCGTCTCAAAGATTAAGCCATGCACGTCTAAGTATAAATATTTTACTTTGAAACTGCGAATGGCTCATTATATCAGTTCTAATTTATTTGATAGTCCCTTACTACTTGGATACCCGTAG
528F+1055R_CNEO-N1
        Consensus  ..................................................................................................................................

                   131     140       150       160       170       180       190       200       210       220       230       240       250       260
                   |--------+---------+---------+---------+---------+---------+---------+---------+---------+---------+---------+---------+---------|
       AM502013.1  TAATTCTAGAGCTAATACATGCGTCAATACCCCTTGTGGGTAGTATTTATTAGATGGAAACCAACCCCTTCGGGGTGATGTGGTGATTCATAATAAGCTTGCGGATCGCATGGCTATGCCGGCGATGGAT
528F+1055R_CNEO-N1
        Consensus  ..................................................................................................................................

                   261     270       280       290       300       310       320       330       340       350       360       370       380       390
                   |--------+---------+---------+---------+---------+---------+---------+---------+---------+---------+---------+---------+---------|
       AM502013.1  CATTCAAGTTTCTGCCCTATCAGCTTTGGATGGTAGGGTATTGGCCTACCATGGCTTTAACGGGTAACGGAGGATTAGGGTTTGATTCCGGAGAGGGGGCCTGAGAGATGGCCACCACATCCAAGGAAGG
528F+1055R_CNEO-N1
        Consensus  ..................................................................................................................................

                   391     400       410       420       430       440       450       460       470       480       490       500       510       520
                   |--------+---------+---------+---------+---------+---------+---------+---------+---------+---------+---------+---------+---------|
       AM502013.1  CAGCAGGCGCGTAAATTACCCAATCCTGACACAGGGAGGTAGTGACAATAAATAACAATGACGGACCCTTTCGGGTCTGTCAATTGGAATGAGAACGATTTAAACCCCCTATCGAGGACCCATTGGAGGG
528F+1055R_CNEO-N1
        Consensus  ..................................................................................................................................

                   521     530       540       550       560       570       580       590       600       610       620       630       640       650
                   |--------+---------+---------+---------+---------+---------+---------+---------+---------+---------+---------+---------+---------|
       AM502013.1  CAAGTCTGGTGCCAGCAGCCGCGGTAATTCCAGCTCCAATAGCGTATATTAAAGTTGTTGCAGTTAAAAAGCTCGTAGTTGGATTTGTGGATGCTCACTTGGTCCTCT-TGAGGCGCCAGTGTGGGGATC
528F+1055R_CNEO-N1                                                        TTGTTGCAGTTAAAAAGCTCGTAGTTGGATTTGTGGTTGCTCACTTGGCCCTCTCTGAGGCGCCAGTGTGGGGATC
        Consensus  ......................................................TTGTTGCAGTTAAAAAGCTCGTAGTTGGATTTGTGGaTGCTCACTTGGcCCTCT.TGAGGCGCCAGTGTGGGGATC

                   651     660       670       680       690       700       710       720       730       740       750       760       770       780
                   |--------+---------+---------+---------+---------+---------+---------+---------+---------+---------+---------+---------+---------|
       AM502013.1  CATCCTTGGGTGGATGCTGTGTGGCATTCGGTTGTCGCGCAGGTGATGCCCATCGTTTACTGTGAAAAAATCAGCGCGTTCAAAGCAGGCTTATGCCGTTGAATGTATTAGCATGGAATAATGAGATAGG
528F+1055R_CNEO-N1 CATCCTTGGGCGGAAGTTGCGTGGCATTCGGTTGTCGCGCAGGGGATGCCCATCGTTTACTGTGAAAAAATCAGCGCGTTCAAAGCAGGCTTATGCCGTTGAATGTATTAGCATGGAATAATGAGATAGG
        Consensus  CATCCTTGGGcGGAaGcTGcGTGGCATTCGGTTGTCGCGCAGGgGATGCCCATCGTTTACTGTGAAAAAATCAGCGCGTTCAAAGCAGGCTTATGCCGTTGAATGTATTAGCATGGAATAATGAGATAGG

                   781     790       800       810       820       830       840       850       860       870       880       890       900       910
                   |--------+---------+---------+---------+---------+---------+---------+---------+---------+---------+---------+---------+---------|
       AM502013.1  ACCTCGGTACTATTTTGTTGGTTTGCGCACTGGGGTAATGATTAATAGGGACAGTTGGGGGTATTCGTATTGCATTGTCAGAGGTGAAATTCTTGGATTTCTGCAAGACGAACTACTGCGAAAGCATTTA
528F+1055R_CNEO-N1 GCCTCGGTACTATTTTGTTGGTTTGCGCACTGGGGTAATGATTAATAGGGACAGTTGGGGGTATTCGTATTGCATTGTCAGAGGTGAAATTCTTGGATTTCTGCAAGACGAACTACTGCGAAAGCATTTA
        Consensus  aCCTCGGTACTATTTTGTTGGTTTGCGCACTGGGGTAATGATTAATAGGGACAGTTGGGGGTATTCGTATTGCATTGTCAGAGGTGAAATTCTTGGATTTCTGCAAGACGAACTACTGCGAAAGCATTTA

                   911     920       930       940       950       960       970       980       990      1000      1010      1020      1030      1040
                   |--------+---------+---------+---------+---------+---------+---------+---------+---------+---------+---------+---------+---------|
       AM502013.1  CCAAGGATGTTTTCATTAATCAAGAACGAAAGTTAGGGGATCGAAGATGATTAGATACCATCGTAGTCTTAACCATAAACTATGCCGACAAGGGATTGGTGGGGT-GTCGTTACGTCTCCATCAGCACCT
528F+1055R_CNEO-N1 CCAAGGATGTTTTCATTAATCAAGAACGAAAGTTAGGGGATCGAAGATGATTAGATACCATCGTAGTCTTAACCATAAACTATGCCGACAAGGGATTGGTGGGGTTATCGTTACGTCTCCATCAGCACCT
        Consensus  CCAAGGATGTTTTCATTAATCAAGAACGAAAGTTAGGGGATCGAAGATGATTAGATACCATCGTAGTCTTAACCATAAACTATGCCGACAAGGGATTGGTGGGGT.aTCGTTACGTCTCCATCAGCACCT

                   1041   1050      1060      1070      1080      1090      1100      1110      1120      1130      1140      1150      1160      1170
                   |--------+---------+---------+---------+---------+---------+---------+---------+---------+---------+---------+---------+---------|
       AM502013.1  TATGAGAAATCACAAGTTTTTGGGTTCCGGGGGGAGTATGGTCGCAAGGCTGAAACTTAAAGAAATTGACGGAAGGGCACCACCAGGAGTGGAGCCTGCGGCTTAATTTGACTCAACACGGGAAAACTTA
528F+1055R_CNEO-N1 TATGAGAAATCACAAGTTTTTGGGTTCCGGGGGGAGTATGGTCGCAAGGCTGAAACTTAAAGAAATTGACGGAAGGGCACCACCAGGAGTGGAGCCTGCGGCTTAATTTGACTCAACACGGGAAAACTCA
        Consensus  TATGAGAAATCACAAGTTTTTGGGTTCCGGGGGGAGTATGGTCGCAAGGCTGAAACTTAAAGAAATTGACGGAAGGGCACCACCAGGAGTGGAGCCTGCGGCTTAATTTGACTCAACACGGGAAAACTcA

                   1171   1180      1190      1200      1210      1220      1230      1240      1250      1260      1270      1280      1290      1300
                   |--------+---------+---------+---------+---------+---------+---------+---------+---------+---------+---------+---------+---------|
       AM502013.1  CCAGGTCCAGACATAGTGAGGATTGACAGATTGAGAGCTCTTTCTTGATTCTATGGTTGGTGGTGCATGGCCGTTCTTAGTTGGTGGAGTGATTTGTCTGGTTAATTCCGTTAACGAACGAGACCGCTGC
528F+1055R_CNEO-N1 CCAGGTCCAGACATAGTGAGGAT-GACAGATTGAGAGTT
        Consensus  CCAGGTCCAGACATAGTGAGGAT.GACAGATTGAGAGcT...........................................................................................

                   1301   1310      1320      1330      1340      1350      1360      1370      1380      1390      1400      1410      1420      1430
                   |--------+---------+---------+---------+---------+---------+---------+---------+---------+---------+---------+---------+---------|
       AM502013.1  CTGCTAAATAGTCCCGCGAGTGATTCTCACTGGCGAGGTCTTCTTAGAGGGACGTGCATTCTATTCGATGCAGGAAGATAGCGGCGATAACAGGTCTGTGATGCCCTTAGATGTTCTGGGCCGCACGCGC
528F+1055R_CNEO-N1
        Consensus  ..................................................................................................................................

                   1431   1440      1450      1460      1470      1480      1490      1500      1510      1520      1530      1540      1550      1560
                   |--------+---------+---------+---------+---------+---------+---------+---------+---------+---------+---------+---------+---------|
       AM502013.1  GCTACACTGATGCATTCAACGAGTTTCCTTGGCCGAGAGGCCTGGGCAATCTTTTGAACGTGCATCGTGATAGGGATAGATTCTTGCAATTATTAATCTTGAACGAGGAATTCCTAGTAAACGCAGATCA
528F+1055R_CNEO-N1
        Consensus  ..................................................................................................................................

                   1561   1570      1580      1590      1600      1610      1620      1630      1640      1650      1660      1670      1680      1690
                   |--------+---------+---------+---------+---------+---------+---------+---------+---------+---------+---------+---------+---------|
       AM502013.1  TCAATCTGCATTGATTACGTCCCTGCCCTTTGTACACACCGCCCGTCGCACCTACCGATTGAATGGTCCGGTGAAGCCTCGAGATTGTGACGTGGGCCTCGGCTCACGGCGCGAGAACTTGTCTAAACCT
528F+1055R_CNEO-N1
        Consensus  ..................................................................................................................................

                   1691   1700      1710      1720    1728
                   |--------+---------+---------+-------|
       AM502013.1  TATCATTTAGAGGAAGGTGAAGTCGTAACAAGGTTTCC
528F+1055R_CNEO-N1
        Consensus  ......................................
```
