# Supplementary material for: Identification of Cocconeis neothumensis var. marina using a polyphasic approach including ultrastructure and gene annotation
Source: PLoS One. 2025 Feb 13;20(2):e0317360. doi: 10.1371/journal.pone.0317360 (PMC11825096; doi:10.1371/journal.pone.0317360)
Supplement: S4 Fig — The sequences of the diatom Cocconeis neothumensis obtained in this work were aligned with the BLAST first hit Cocconeis euglypta (Accession Number: LR890021.1) exhibiting 94.6% of pairwise sequence similarity. (PDF) [file pone.0317360.s004.pdf]

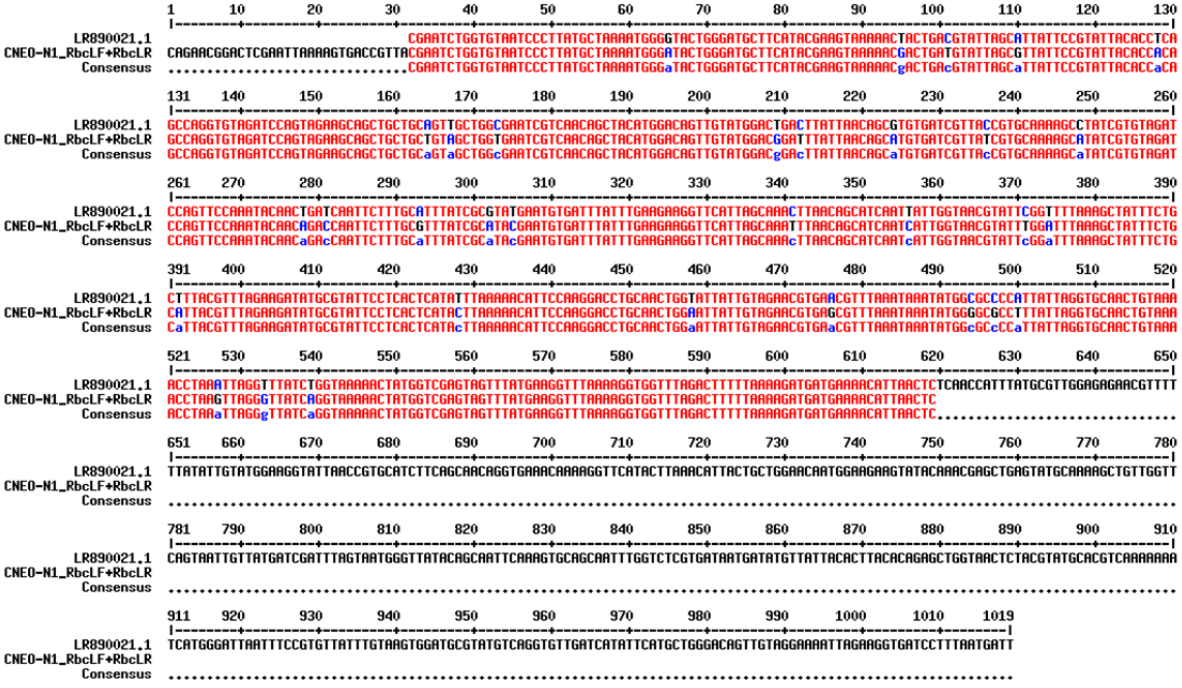
1 10 20 30 40 50 60 70 80 90 100 110 120 130
LR890021.1 CGAATCTGGTGTAATCCCTTATGCTAAAATGGGGTACTGGGATGCTTCATACGAAGTAAAAACTACTGACGTATTAGCATTATTCCGTATTACACCTCA
CNEO-N1_RbcLF+RbcLR CAGAACGGACTCGAATTAAAAGTGACCGTTACGAATCTGGTGTAATCCCTTATGCTAAAATGGGATACTGGGATGCTTCATACGAAGTAAAAACGACTGATGTATTAGCGTTATTCCGTATTACACCACA
Consensus .....CGAATCTGGTGTAATCCCTTATGCTAAAATGGGaTACTGGGATGCTTCATACGAAGTAAAAACgACTGAcGTATTAGCaTTATTCCGTATTACACCaCA
131 140 150 160 170 180 190 200 210 220 230 240 250 260
LR890021.1 GCCAGGTGTAGATCCAGTAGAAGCAGCTGCTGCAGTTGCTGGCGAATCGTCAACAGCTACATGGACAGTTGTATGGACTGACTTATTAACAGCGTGTGATCGTTACCGTGCAAAAGCCTATCGTGTAGAT
CNEO-N1_RbcLF+RbcLR GCCAGGTGTAGATCCAGTAGAAGCAGCTGCTGCTGTAGCTGGTGAATCGTCAACAGCTACATGGACAGTTGTATGGACGGATTTATTAACAGCATGTGATCGTTATCGTGCAAAAGCATATCGTGTAGAT
Consensus GCCAGGTGTAGATCCAGTAGAAGCAGCTGCTGCaGTaGCTGGcGAATCGTCAACAGCTACATGGACAGTTGTATGGACgGAcTTATTAACAGCaTGTGATCGTTAcCGTGCAAAAGCaTATCGTGTAGAT
261 270 280 290 300 310 320 330 340 350 360 370 380 390
LR890021.1 CCAGTTCCAAATACAACTGATCAATTCTTTGCATTTATCGCGTATGAATGTGATTTATTTGAAGAAGGTTCATTAGCAAACTTAACAGCATCAATTATTGGTAACGTATTCGGTTTTAAAGCTATTTCTG
CNEO-N1_RbcLF+RbcLR CCAGTTCCAAATACAACAGACCAATTCTTTGCGTTTATCGCATACGAATGTGATTTATTTGAAGAAGGTTCATTAGCAAATTTAACAGCATCAATCATTGGTAACGTATTTGGATTTAAAGCTATTTCTG
Consensus CCAGTTCCAAATACAACaGAcCAATTCTTTGCaTTTATCGCaTAcGAATGTGATTTATTTGAAGAAGGTTCATTAGCAAAcTTAACAGCATCAATcATTGGTAACGTATTcGGaTTTAAAGCTATTTCTG
391 400 410 420 430 440 450 460 470 480 490 500 510 520
LR890021.1 CTTTACGTTTAGAAGATATGCGTATTCCTCACTCATATTTAAAAACATTCCAAGGACCTGCAACTGGTATTATTGTAGAACGTGAACGTTTAAATAAATATGGCGCCCCATTATTAGGTGCAACTGTAAA
CNEO-N1_RbcLF+RbcLR CATTACGTTTAGAAGATATGCGTATTCCTCACTCATACTTAAAAACATTCCAAGGACCTGCAACTGGAATTATTGTAGAACGTGAGCGTTTAAATAAATATGGGGCGCCTTTATTAGGTGCAACTGTAAA
Consensus CaTTACGTTTAGAAGATATGCGTATTCCTCACTCATAcTTAAAAACATTCCAAGGACCTGCAACTGGaATTATTGTAGAACGTGAaCGTTTAAATAAATATGGcGCcCCaTTATTAGGTGCAACTGTAAA
521 530 540 550 560 570 580 590 600 610 620 630 640 650
LR890021.1 ACCTAAATTAGGTTTATCTGGTAAAAACTATGGTCGAGTAGTTTATGAAGGTTTAAAAGGTGGTTTAGACTTTTTAAAAGATGATGAAAACATTAACTCTCAACCATTTATGCGTTGGAGAGAACGTTTT
CNEO-N1_RbcLF+RbcLR ACCTAAGTTAGGGTTATCAGGTAAAAACTATGGTCGAGTAGTTTATGAAGGTTTAAAAGGTGGTTTAGACTTTTTAAAAGATGATGAAAACATTAACTC
Consensus ACCTAAaTTAGGgTTATCaGGTAAAAACTATGGTCGAGTAGTTTATGAAGGTTTAAAAGGTGGTTTAGACTTTTTAAAAGATGATGAAAACATTAACTC.....
651 660 670 680 690 700 710 720 730 740 750 760 770 780
LR890021.1 TTATATTGTATGGAAGGTATTAACCGTGCATCTTCAGCAACAGGTGAAACAAAAGGTTCATACTTAAACATTACTGCTGGAACAATGGAAGAAGTATACAAACGAGCTGAGTATGCAAAAGCTGTTGGTT
CNEO-N1_RbcLF+RbcLR
Consensus .....
781 790 800 810 820 830 840 850 860 870 880 890 900 910
LR890021.1 CAGTAATTGTTATGATCGATTTAGTAATGGGTTATACAGCAATTCAAAGTGCAGCAATTTGGTCTCGTGATAATGATATGTTATTACACTTACACAGAGCTGGTAACTCTACGTATGCACGTCAAAAAAA
CNEO-N1_RbcLF+RbcLR
Consensus .....
911 920 930 940 950 960 970 980 990 1000 1010 1019
LR890021.1 TCATGGGATTAATTTCCGTGTTATTTGTAAGTGGATGCGTATGTCAGGTGTTGATCATATTCATGCTGGGACAGTTGTAGGAAAATTAGAAGGTGATCCTTTAATGATT
CNEO-N1_RbcLF+RbcLR
Consensus .....
